# Human development II: We Need an Integrated Theory for Matter, Life and Consciousness to Understand Life and Healing

**DOI:** 10.1100/tsw.2006.154

**Published:** 2006-07-06

**Authors:** Sören Ventegodt, Tyge Dahl Hermansen, Maj Lyck Nielsen, Birgitte Clausen, Joav Merrick

**Affiliations:** ^1^ Quality of Life Research Center, Teglgårdstræde 4-8, DK-1452 Copenhagen K, Denmark; ^2^ Research Clinic for Holistic Medicine, Copenhagen, Denmark; ^3^ Nordic School of Holistic Medicine, Copenhagen, Denmark; ^4^ Scandinavian Foundation for Holistic Medicine, Sandvika, Norway; ^5^ Interuniversity College, Graz, Austria; ^6^ Vejlby Lokalcenter, Vejlby, Denmark; ^7^ National Institute of Child Health and Human Development, Jerusalem, Israel; ^8^ Center for Disability and Human Development, Faculty of Health Sciences, Ben Gurion University, Beer-Sheva, Israel; ^9^ Office of the Medical Director, Division for Mental Retardation Ministry of Social Affairs, Jerusalem, Israel

**Keywords:** quality of life, QOL, holistic biology, clinical holistic medicine, public health, theoretical biology, metamorphosis, information-directed self-organization, cosmology, Denmark

## Abstract

For almost a decade, we have experimented with supporting the philosophical development of severely ill patients to induce recovery and spontaneous healing. Recently, we have observed a new pattern of extremely rapid, spontaneous healing that apparently can facilitate even the spontaneous remission of cancer and the spontaneous recovery of mental diseases like schizophrenia and borderline schizophrenia. Our working hypothesis is that the accelerated healing is a function of the patients brain-mind and body-mind coming closer together due to the development of what we call “deep” cosmology. To understand and describe what happens at a biological level, we have suggested naming the process *adult human metamorphosis*, a possibility that is opened by the human genome showing full generic equipment for metamorphosis. To understand the mechanistic details in the complicated interaction between consciousness and biology, we need an adequate theory for biological information. In a series of papers, we propose what we call “holistic biology for holistic medicine”. We suggest that a relatively simple model based on interacting wholenesses instead of isolated parts can shed a new light on a number of difficult issues that we need to explain and understand in biology and medicine in order to understand and use metamorphosis in the holistic medical clinic. We aim to give a holistic theoretical interpretation of biological phenomena at large, morphogenesis, evolution, immune system regulation (self-nonself discrimination), brain function, consciousness, and health in particular. We start at the most fundamental problem: what is biological information at the subcellular, cellular, and supracellular levels if we presume that it is the same phenomenon on all levels (using Occam's razor), and how can this be described scientifically? The problems we address are all connected to the information flow in the functioning, living organism: function of the brain and consciousness, the regulations of the immune system and cell growth, the dynamics of health and disease. We suggest that life utilizes an unseen fine structure of the physical energy of the universe at a subparticular or quantum level to give information-directed self-organization; we give a first sketch of a possible fractal structure of the energy able to both contain and communicate biological information and carry individual and collective consciousness. Finally, thorough our analysis, we put up a model for adult human metamorphosis.

## INTRODUCTION

Recently, we have observed what seems to be a completely new pattern of highly accelerated, spontaneous healing in the holistic medical clinic where we help people to improve their quality of life, health, and ability by developing their consciousness and the value they create by using more of their inborn talents. The new pattern of healing resembles the process of metamorphosis of the caterpillar/butterfly —the larvae turns into pupae and, in only 10 days, melts totally down to reappear as the adult butterfly. We have noticed that the human genome contains full equipment for metamorphosis and actually very much the same genes for metamorphosis as insects and lower vertebrates, like the amphibian. The frog goes through a radical metamorphosis from tadpole to frog, and we were frogs not long ago on the evolutionary scale. The genes for human metamorphosis are known to be active during ontogenesis, but we have observed adult human metamorphosis[1]. We have seen the change in the whole character of the person, from purpose of life, to cognitive patterns of the interpretation of the world, to the actual physical form of the body. The patients that enter metamorphosis seem to have two things in common: (1) they have been arrested in their psychosexual development early in life, often due to severe sexual or violent abuse or neglect, and (2) they have come to a sudden deeper understanding of life. This deeper understanding must be of the form we would call “a deepening of their cosmology”.

We have observed that our understanding of the world can go through evolution and revolution. The evolution is the gradual growth of insight and understanding, and the revolution is a crisis, where everything loses its normal meaning and significance in a way, and in this void, a completely new fundamental pattern of understanding of the world appears. What seems to happen is that the structure of the conceptual tree in the brain-mind changes; in our analysis, this structure is fractal and what happens is that the bifurcation number changes[2]. When it goes down, we run away from our existential responsibility and this event often seems to happen in our early childhood, where responsibility becomes too painful[3]. When the adult person searches for deeper understanding, this reduced cosmology relates[4], so survival is re-exchanged with a deeper, more complex cosmology, looking very much like the original “natural” and deep, emotionally rich cosmology of the small child[4]. Interestingly, the brain-mind and the body-mind (the informational system of the organism) seem to be separated by the cosmology going down in number, separating also mind and body, and making emotions less intense[4]. As an adult, the opposite happens: a new and deeper cosmology appears, a new emotional and experiential life being, with more intense living, more focus on the body, on enjoying, on sexuality, and on existence[4]. So it seems that deepening the cosmology is a hallmark of existential healing. It seems that this healing brings the brain-mind and the body-mind together, making the person more whole and more emotional. When body and mind merge, the totality of the being surfaces, and the self is perceived more transcendentally. We say that the person recovers the existential coherence, because life inside is reconnected with the world outside and quality of life (QOL), health (both mental and physical), and all the abilities of the person are often radically improved.

Adult human metamorphosis is strange in that this process happens in weeks, not over several years, as normal therapy does. The patient undergoing metamorphosis often becomes completely introverted and enters a highly visionary state of existence, known from Native American rituals like the Mitote (using the hallucinogenic cactus peyote[5]) and the Vision Quest (using no drugs, but focused intent on transformation[6]). More peculiar is the very ill patient's tendency to recover suddenly, or so it seems, from even cancer[7,8,9] and schizophrenia[10,11], when QOL improved during and after transformation.

What we have found is highly unexpected and it seems to call on radical new thinking; it simply needs a new, more holistic, theory of biology and the role of consciousness both in ontogenesis and phylogenesis. As we see it, it calls for a deep understanding of biological information, for the informational flows and the appearance of the hidden person almost in one magical stroke is really what needs explanation. Of course, we already know metamorphosis from the animal world, so it cannot be that much of a shock for the observant reader to see human beings melting down, entering the state of the human pupae, and reappearing as a completely upgraded version of themselves after a few weeks. Even recovering from severe mental or somatic disease is really unexpected, even for the most open-minded physician.

## OUR CLINICAL EXPERIENCE AND OBSERVATIONS

It might be that we are completely mistaken or mistaken in our interpretations of what we observe. On the other hand, during the last 14 years, we have been studying human development first, then the phenomenon of holistic healing, then human transformation, and now the accelerated process of human metamorphosis, which seems also to explain the slower processes of spontaneous healing in biological terms.

It is quite clear from studying the patients who enter the transitional state of metamorphosis that the patients seem to open up to what Carl Gustav Jung used to call "the collective consciousness"[12]: a layer of immense information about being human, that all human beings can dive into for inspiration and renewal. We have learned just recently that many premodern cultures like the Native American cosmology of the Delicate Lodge — in our analysis carrying the bifurcation-number 8 (an "8-ray cosmology") — in its ontology has the collective consciousness of plants (called "The White Buffalo Woman"), animals (called "Sacred Sweet Medicine"), and man. So we know that different people and cultures have appreciated these deep information-carrying levels of human existence. Western medicine is just now opening up to their existence in research[13,14].

Professor Daniel Shek from Hong Kong (personally communication) has talked about "the three Ts of holistic medicine: transformation, transcendence, and totality". This is very accurately the focus of our holistic medical endeavor of healing our patients, which are not only the traditionally ill patients, but everybody that needs an improvement of QOL. The accelerated human growth of metamorphosis also seems to be very promising for helping the most troubled and harmed of the people.

## HOLISTIC UNDERSTANDING OF LIFE

Our interest in theoretical biology started in the 1980s, but for 2 decades, our work seemed to be too idiosyncratic to be published. Only now, after first testing the models and noticing their positive consequences for holistic medicine, have we gained sufficient confidence in these models to publish them. The induction of spontaneous healing from unsolvable health problems, like cancer and severe mental diseases, at our Research Clinic for Holistic Medicine[7,8,9,10] for almost a decade has also given us the belief that it is time to publish our unconventional perspective on biology and human development. We have recently presented the first ideas of holistic biology[15,16].

The idiosyncratic feeling stems from the radical belief that we need to go all the way down to the fundamental energy of the universe to understand biology. Instead of using established mathematical structures as the basis of our model, in 1985 we looked for a more intuitive, correct presentation of biological informational systems of the living organisms, from single cells to large ecosystems. Unfortunately, the structures that followed from this quest were not compatible with any known mathematical structures at that time. As you might have guessed, we discovered, as did Benoit B Mandelbrot and many others at that time, the fractal nature of the living world. We made our first fractal model for the organism in 1986[2]. After 20 years, everybody seems to have accepted the fractals, but biology has not been better explained after all. So we are still missing the point. What we try to bring forward now is what we believe to be the missing parts of the puzzle.

Our major point is that the well-known order of the universe described by contemporary science is illusory in a way, and our certainty of many things might make us overlook a hidden order in the universe, which guides not only how the subatomic energy and particles are organized into atoms and molecules[17], but also how matter is organized into the living cell, with its dynamic organelles and highly advanced supramolecular structures[18]. It might be the same principles that guide the cells into a complex organism[19], and the organisms into extremely complex and "intelligently" created biospheres, including planet Earth at large[20]. And it might even be the same principles that count for "white" and dark matter organizing the dust, planets, and stars into galaxies and clusters of galaxies.

## OUR SUGGESTIONS

We suggest that there exists one hidden, but highly active, fundamental, organizing principle that we call *the fine structure of the physical energy*, and we believe that a first approximation to a description of this energy is a spiral fractal[2]. We suggest a simple geometry in which all levels interact with each other and with every part existing on each level. Unfortunately, this geometry has been difficult to formalize. As with most other fractals, it cannot be built on logic and set theory. Interestingly, the description we need is fundamentally different from the principles of logic and the sets of the set theory, as both logic and the set theory are based on the idea of a well-defined element distinctly separated from its surroundings, in that it has qualities that are unique to this element and not dependent on the state of the space or of other coexisting elements. Our normal logic only has meaning if such elements exist. The "dependent" elements of the real world have, as you might know from quantum mechanics, complex and mysterious qualities that have been extremely difficult to define.

Our fundamental postulate that started our thinking is that *independent elements do not really exist.* In the world of nonliving "dead" objects, we know that independent elements do not exist on the level of the particle, but large dead elements seem to function almost independent of each other (one is red and square, another blue and round), and researchers for many years have believed that large living organisms could be understood in parallel to large dead objects in this respect.

Our position is that living beings have been specialized in using the deep coherence and connectedness of everything, and that consciousness has risen from this connectedness. The coherence of living organisms might be important for evolution. It could help the animal of a species to develop simultaneously (when one fly gets its wings and becomes successful this way, the other flies are encouraged through this connectedness also to turn winged[20]).

We also believe the single cell to be conscious (as do other thinkers in the field: Ben Jacob, personal communication, and [21]) and able to make decisions on its own, changing its behaviors or other characteristics of the whole cell. We also believe that the consciousness of the whole organism rises from the multiple consciousnesses of its cells[12]. These cells have the same ability to cohere as all living beings, and they use this coherence to get information to make valuable decisions and thus change their own character and also the character of the whole organism. The cells are efficiently using the fundamental coherent nature of the world, which is so deeply mysterious that we do not have a theory for it yet. This is the theory we want to make.

We believe that science needs a completely new language to describe and analyze what is happening in the living world, and the present work is a first try to provide such a language. We hope that the solution we have found will shed light on many of the unsolved problems of biology. Most interestingly, many of the human diseases and the patient's spontaneous healing seem to be understandable on top of this new understanding of biology, the body, immunology, the human brain, and consciousness.

## MAY THIS WORK BE OF VALUE TO ALL LIVING

What we want to model is the internal and mechanistic structure of this coherence of the elements and beings of the living world on the many levels of the biological universe, from molecule over the organelle and cell to the whole organism, its family, its species and the ecosystem it inhabits. The phenomenon we have identified seems to be a principle almost only used by living organisms. We have called it "*information-directed self-organization*". We have analyzed a number of biological systems and situations, and we have found that the concept of "information-directed self-organization" can explain yet unexplained biological phenomena.

Most interestingly, the new symbolic language seems to be able to explain what wholeness is. For instance: man is a wholeness, living in a wholeness of the world. We all feel our autonomous freedom to be, do, and think. But are we free to make choices? Are we free to perceive? Are we determined of our history and our world, or are we really causal to our world? Can we create our own life, healthy and happy, independently of the surrounding world and our personal past, gene pool, etc.? Can we change our own destiny and get well again even from metastatic cancer? Can we go through metamorphosis and change both mind and body, purpose of life, and our fundamental experience of the world – our worldview or cosmology? We believe that we can. But where is then the theoretical limit for our human abilities? It is all such questions that we want to answer and this is the quest that has motivated the present analysis of life and man.

## ACKNOWLEDGMENTS

These studies were supported by grants from IMK Almene Fond. Our research in quality of life has been approved by the Copenhagen Scientific Ethical Committee under number (KF)V.100.2123/91.

## References

1. Ventegodt, S., Hermansen, T.D., Clausen, B., Nielsen, M.L., and Merrick, J. (2006) Human development XX: a theory for spontaneous healing: adult human metamorphosis. Submitted to *TheScientificWorldJOURNAL*.
2. Ventegodt, S., Hermansen, T.D., Flensborg-Madsen, T., Rald, E., Nielsen, M.L., Clausen, B., and Merrick, J. (2006) Human development VII: a spiral fractal model of fine structure of physical energy could explain central aspects of biological information, biological organisation, and biological creativity. Submitted to *TheScientificWorldJOURNAL*
3. Ventegodt, S., Hermansen, T.D., Rald, E., Clausen, B., Nielsen, M.L., and Merrick, J. (2006) Human development XIV: degeneration and regeneration of existence – metamorphosis changes the purpose of life, the level of existential responsibility, and the depth of consciousness (the ray-number of the cosmology). Submitted to *TheScientificWorldJOURNAL*.
4. Ventegodt, S., Kromann, M., Andersen, N.J., and Merrick, J. (2004) The life mission theory VI. A theory for the human character: healing with holistic medicine through recovery of character and purpose of life. *TheScientificWorldJOURNAL* **4**, 859–880.
5. Anderson, E.F. (1996) *Peyote: The Divine Cactus.* University of Arizona Press.
6. Moondance, W. and Starhawk, S. (2004) *Vision Quest: Native American Magical Healing.* Sterling.
7. Spiegel, D., Bloom, J.R., Kraemer, H.C., and Gottheil, E. (1989) Effect of psychosocial treatment on survival of patients with metastatic breast cancer. *Lancet* **2(8668)**, 888–891.
8. Ventegodt, S., Morad, M., and Merrick, J. (2004) Clinical holistic medicine: Induction of Spontaneous Remission of Cancer by Recovery of the Human Character and the Purpose of Life (the Life Mission). *TheScientificWorldJOURNAL* **4**, 362–377.
9. Ventegodt, S., Solheim, E., Saunte, M.E., Morad, M., Kandel, I., and Merrick, J. (2004) Clinical holistic medicine: metastatic cancer. *TheScientificWorldJOURNAL* **4**, 913–935.

10. Ventegodt, S., Andersen, N.J., Neikrug, S., Kandel, I., and Merrick, J. (2005) Clinical holistic medicine: mental disorders in a holistic perspective. *TheScientificWorldJOURNAL* **5**, 313–323.
11. Ventegodt, S., Andersen, N.J., Neikrug, S., Kandel, I., and Merrick, J. (2005) Clinical holistic medicine: holistic treatment of mental disorders. *TheScientificWorldJOURNAL* **5**, 427–445.
12. Hermansen, T.D., Ventegodt, S., Clausen, B., Nielsen, M.L., and Merrick, J. (2006) Human development VIII: a theory of "deep" quantum chemistry and cell consciousness. A deeply structured quantum field can give cells memory, coherence, intent, consciousness, and choice. Submitted to *TheScientificWorldJOURNAL*.
13. Grof, S. (2000) *Psychology of the Future: Lessons from Modern Consciousness Research.* SUNY Series in Transpersonal and Humanistic Psychology. State University of New York Press.
14. Wilber, K. (2000) *Integral Psychology: Consciousness, Spirit, Psychology, Therapy.* Shambhala.
15. Ventegodt, S., Merrick, J., and Andersen, N.J. (2003) QOL philosophy III: towards a new biology: understanding the biological connection between quality of life, disease, and healing. *TheScientificWorldJOURNAL* **3**, 1186–1198.
16. Ventegodt, S., Andersen, N.J., and Merrick, J. (2003) QOL philosophy IV: the brain and consciousness. *TheScientificWorldJOURNAL* **3**, 1199–1209.
17. Ventegodt, S., Hermansen, T.D., Rald, E., Flensborg-Madsen, T., Nielsen, M.L., Clausen, B., and Merrick, J. (2006) Human development III: bridging brain-mind and body-mind. Introduction to "deep" (fractal, poly-ray) cosmology. Submitted to *TheScientificWorldJOURNAL*.
18. Ventegodt, S., Hermansen, T.D., Flensborg-Madsen, T., Clausen, B., Nielsen, M.L., and Merrick, J. (2006) Human Development: the living cell has information-directed self-organisation – and consciousness. Submitted to *TheScientificWorldJOURNAL*
19. Ventegodt, S., Hermansen, T.D., Flensborg-Madsen, T., Clausen, B., Nielsen, M.L., and Merrick, J. (2006) Human Development: supra-cellular morphogenesis - the origin of biological and cellular order. Submitted to *TheScientificWorldJOURNAL.*
20. Hermansen, T.D., Ventegodt, S., Clausen, B., Nielsen, M.L., and Merrick, J. (2006) Human Development: explanation of the macro-evolution: top down evolution materialises consciousness. The origin of metamorphosis. Submitted to *TheScientificWorldJOURNAL.*
21. Penrose, R. and Gardner, M. (2002) *The Emperor's New Mind: Concerning Computers, Minds, and the Laws of Physics*. Oxford University Press.

## BIOSKETCHES

**Søren Ventegodt, MD,** is the director of the Nordic School of Holistic Health and Quality of Life Research Center in Copenhagen, Denmark. He is also responsible for a Clinical Research Clinic for Holistic Medicine in Copenhagen and is a popular speaker throughout Scandinavia. He has published numerous scientific or popular articles and a number of books on holistic medicine, quality of life, and quality of working life. His most important scientific contributions are the comprehensive SEQOL questionnaire, the very short QOL5 questionnaire, the integrated QOL theory, the holistic process theory, the life mission theory, and the ongoing Danish Quality of Life Research Survey, 1991–94 in cooperation with the University Hospital of Copenhagen and the late professor of pediatrics, Bengt Zachau-Christiansen, MD, PhD. E-mail: ventegodt@livskvalitet.org. Website: www.livskvalitet.org

**Tyge Dahl Hermansen**, **MSc** in biology, is a research assistant at the Quality of Life Research Center, Copenhagen. Research areas include molecular biology, physiology, and life quality; experimental experience in fruit flies, yeast, bacteria, and cnidarians; theoretical research in human life quality; technical knowledge in the areas of animal physiology, molecular biology, biotechnology, biochemistry, cell biology, and immunology. Publications appear in popular scientific as well as international journals. E-mail: TD@livskvalitet.org

**Maj Lyck Nielsen,** Occupational Therapist, specialized in psychiatry, Holistic Therapy assistant at the Quality of Life Research Center in Copenhagen, Denmark. Student at the Nordic School of Holistic Medicine. E-mail: mln@livskvalitet.org

**Birgitte Clausen, RN,** leader of a secluded ward for people with dementia. Dementia coordinator and consultant in Aarhus, Denmark with more than 10 years of experience in adult psychiatry and dementia. During the last 4 years, she has worked together with Søren Ventegodt on the "QOL as medicine project" and on developing existential holistic group therapy. Through her articles and lectures, she has disseminated "The New Culture of Dementia" in both Denmark and Japan. She has participated in the production of several videos to order to inspire relatives, medical staff, and volunteers to understand people with dementia. This project has been granted financial support from the Ministry of Social Affairs in Denmark. She has conducted the first Danish investigation on the effect of sufficient daylight on the human spirit, activity level, and biological rhythm. E-mail: villahvide@mail.dk

**Joav Merrick, MD, MMedSci, DMSc,** is professor of child health and human development affiliated with the Center for Multidisciplinary Research in Aging, Zusman Child Development Center, Division of Pediatrics and Community Health at the Ben Gurion University, Beer-Sheva, Israel; the medical director of the Division for Mental Retardation, Ministry of Social Affairs, Jerusalem; and the founder and director of the National Institute of Child Health and Human Development. Dr. Merrick has numerous publications in the field of child health and human development, rehabilitation, intellectual disability, disability, health, welfare, abuse, advocacy, quality of life, and prevention and received the Peter Sabroe Child Award for outstanding work on behalf of Danish Children in 1985 and the International LEGO-Prize ("The Children's Nobel Prize") for an extraordinary contribution towards improvement in child welfare and well being in 1987. E-mail: jmerrick@internet-zahav.net. Website: www.nichd-israel.com
